# TRIP13 Induces Nedaplatin Resistance in Esophageal Squamous Cell Carcinoma by Enhancing Repair of DNA Damage and Inhibiting Apoptosis

**DOI:** 10.1155/2022/7295458

**Published:** 2022-05-10

**Authors:** Lin-Ting Zhang, Li-Xin Ke, Xin-Yi Wu, Hui-Ting Tian, Hua-Zhen Deng, Li-Yan Xu, En-Min Li, Lin Long

**Affiliations:** ^1^Department of Biochemistry and Molecular Biology, Shantou University Medical College, Shantou, 515041 Guangdong Province, China; ^2^Institute of Oncologic Pathology, Shantou University Medical College, Shantou, 515041 Guangdong Province, China; ^3^Institute of Basic Medical Science, Cancer Research Center, Shantou University Medical College, Shantou, 515041 Guangdong Province, China; ^4^Guangdong Provincial Key Laboratory of Infectious Diseases and Molecular Immunopathology, Shantou University Medical College, Shantou, 515041 Guangdong Province, China; ^5^The Key Laboratory of Molecular Biology for High Cancer Incidence Coastal Chaoshan Area, Shantou University Medical College, Shantou, 515041 Guangdong Province, China

## Abstract

Thyroid hormone receptor interactor 13 (TRIP13) plays a crucial role in poor prognosis and chemotherapy resistance of cancer patients. This present study is aimed at investigating the role of high expression of TRIP13 inducing nedaplatin (NDP) resistance in esophageal squamous cell carcinoma (ESCC) cells. High expression of TRIP13 promoted the proliferation and migration of ESCC cells performed by MTS assay, colony formation assay, wound healing assay, and transwell assay. High TRIP13 expression induced NDP resistance to ESCC based on the cell proliferation promoting/inhibition rate and cell migration promoting/inhibition rate analysis, flow cytometry assay of apoptotic subpopulations with a combination of Annexin V-FITC and propidium iodide, and Western blot analysis downregulating cleaved PARP, *γ*H2A.X, cleaved caspase-3, and Bax and upregulating Bcl-2 expression. This study indicated that high expression of TRIP13 promoted proliferation and migration of ESCC cells and induced NDP resistance via enhancing repair of DNA damage and inhibiting apoptosis. This will provide a preliminary reference for the clinical use of NDP in ESCC treatment.

## 1. Introduction

Esophageal squamous cell carcinoma (ESCC) is one of the common malignant tumors with high morbidity and fatality [1]. It remains the predominant pathological type in areas with a high incidence of esophageal cancer in China [1]. At present, surgery is the main treatment strategy for ESCC. However, due to the insidious early symptoms, most of the patients have already progressed to the middle or late stages of the disease at the time of consultation, missing the best time for surgical treatment, and the prognosis for surgery alone is really poor [2]. Therefore, radiotherapy and chemotherapy are often combined for treatment [3–6]. Chemotherapy is contributed to enhance the local control of the tumor, so it is important and necessary to explore the chemotherapeutic drugs for ESCC [7].

There are many kinds of chemotherapeutic drugs, among which platinum drugs are commonly used [8]. Cisplatin, as one of platinum drugs, is recommended as a chemotherapy drug for treating ESCC [8–11]. But cisplatin has lots of toxic side effects, such as nephrotoxicity, peripheral neurotoxicity, and gastrointestinal side effects, which limits the usage [8]. Nedaplatin (NDP), as a derivative of cisplatin, is used widely and recommended for ESCC treatment in Japan [12]. It has fewer side effects than cisplatin except for bone marrow toxicity [9]. However, some kinds of cancers, such as nasopharynx cancer, non-small-cell lung cancer, and cervical cancer, have shown NDP resistance in the previous studies [13–15]. Also, there is cross-resistance between NDP and cisplatin while cisplatin resistance is present in ESCC [16, 17]. The mechanisms of resistance in different tumors are different [18–21]. Therefore, studies for NDP drug resistance in cancers are necessary.

Recent studies have indicated that thyroid hormone receptor interactor 13 (TRIP13) is abnormally expressed in many tumor tissues and related to poor prognosis [21–23]. Interestingly, abnormal expression of TRIP13 in ESCC has been recently noted [23]. In addition, TRIP13 promotes drug resistance, including head and neck squamous cell carcinoma and bladder cancer [21, 22]. However, whether ESCC exists, nedaplatin resistance and its molecular mechanisms underlying its dysregulation remain unclear.

In this study, resistance of ESCC to NDP and its correlation were detected by constructing ESCC cells with abnormal expression of TRIP13 and NDP intervention. The results showed that high expression of TRIP13 could promote the proliferation and migration ability of ESCC cells and contributed to the NDP resistance via enhancing repair of DNA damage and inhibiting apoptosis. This study predicted that TRIP13 could be used as a new therapeutic target for ESCC, laying a foundation and providing reference for the clinical use of NDP in the treatment of ESCC.

## 2. Materials and Methods

### 2.1. Cell Lines and Cell Culture

Human esophageal squamous cell carcinoma cell line TE1 was cultured in DMEM medium (Hyclone, Los Angeles, CA, USA). KYSE150 was cultured in RPMI-1640 medium (Invitrogen, Carlsbad, CA, USA). TE3, KYSE30, and KYSE510 were cultured in Gibco RPMI-1640 medium (Gibco, Grand Island, NY, US). For detailed information about the cells, please refer to the literature previously published in our laboratory [24–26]. The medium contained 10% FBS (Thermo Fisher Scientific, Waltham, MA, USA), and the cultural environment was 5% CO_2_, at 37°C and 80% humidity.

### 2.2. siRNA and Plasmid Transfection

The siRNA sequence of *TRIP13* gene was synthesized by GenePharma (Shanghai, China) (Table 1). The siRNA was dissolved in DEPC water diluted to 20 *μ*M, storing at -20°C for standby use according to the instruction. Human *TRIP13* gene ORF cDNA clone expression plasmid was purchased from Sino Biological (Beijing, China), and *TRIP13* Gene primer (Supplementary Table 1) was purchased from Tianyi Huiyuan (Guangzhou, China). The expression vector was constructed to obtain pcDNA3.1-HA-TRIP13 plasmid and pEGFP-C1-TRIP13 plasmid by Trans5*α* Chemically Competent Cell (Transgen, Beijing, China) with ampicillin (100 mg/ml) (Maygene, Guangzhou, China) and kanamycin (10 mg/ml) (Maygene, Guangzhou, China), according to Biomiga EZgene™ Plasmid Miniprep Kit (Biomiga, Santiago, CA, USA) instruction and Biomiga EZgene™ PCR/Gel Extraction Kit (Biomiga, Santiago, CA, USA) instruction. We use Lipofectamine 3000 (Invitrogen, Shanghai, China) in accordance with the instruction of manufacturers to configure transfection solution and add fresh medium and mix evenly. The medium was changed after 6 h, and the cells were continued to incubate for 24 h.

### 2.3. RNA Extraction and Quantitative Real-Time PCR (qRT-PCR)

Total RNA was extracted by using TRIzol (Invitrogen, Carlsbad, CA, USA). Total RNA was reversely transcribed into cDNA according to HiScript® III RT SuperMix for qPCR (+gDNA wiper) (Vazyme, Piscataway, NJ, USA) instruction, and quantitative PCR was detected according to ChamQ™ Universal SYBR qPCR Master Mix (Vazyme, Piscataway, NJ, USA) instruction. The amplification curve and melting curve of qPCR were obtained based on *β*-actin primer (Supplementary Table 1), and the relative changes of gene expression were analyzed by the 2^-△△CT^ method [25, 26].

### 2.4. Western Blot Analysis

Western blot was used to explore the protein expression level of TRIP13, PARP, cleaved PARP, *γ*H2A.X, caspase-3, cleaved caspase-3, Bcl-2, and Bax [25, 26]. RIPA lysate (Proteintech Group Inc., Chicago, IL, USA), containing PMSF, was used to extract cell total protein. BCA Protein Assay Kit (Pierce; Thermo Fisher Scientific Inc., Waltham, MA, USA) was used to quantify the protein concentration. SDS-PAGE gels were prepared. Proteins were separated using electrophoresis with 45 V (40 min), 60 V (60 min), 80 V (60 min), and 100 V (20-60 min) (stopped according to electrophoresis distance). After electrophoresis, proteins were transferred onto polyvinylidene fluoride (PVDF) membranes with 60 V 2 h. Once the proteins were transferred onto the membranes, membranes would be incubated at room temperature for 1 h with confining liquid (skim milk powder : TBST = 1 : 20) for blocking. Membranes were incubated with primary antibodies on a shaker overnight at 4°C. Then, samples were washed with 1 × TBST 3 times. The corresponding secondary antibody was added for incubation at room temperature for 1.5 h; then membranes were washed 3 times. After that, membranes were fluorescence imaged by multifunctional imaging analysis system (Fluor ChemR 8.3-megapixel CCD, ProteinSimple, Silicon Valley, CA, USA). Specific primary antibodies are as follows: TRIP13 (A-7) (Santa Cruz Biotechnology, Inc., Santa Cruz, CA, USA, 1 : 500), GAPDH (Proteintech, Group, Inc., Chicago, IL, USA, 1 : 5000), and HA (Proteintech Group, Inc., Chicago, IL, USA, 1 : 10000). GFP, *γ*H2A.X, Bcl-2, and Bax are all from the same manufacturer (Santa Cruz Biotechnology, Inc., Santa Cruz, CA, USA, 1 : 1000). PARP (Product# 9542), caspase-3 (Product# 14220T), and cleaved caspase-3 (Product# 9664T) (Cell Signaling Technology, Danvers, MA, USA, 1 : 1000). Specific secondary antibodies are as follows: HRP-conjugated Affinipure Goat Antibody Mouse IgG (H+L) (Proteintech Group, Inc., Chicago, IL, USA, 1: 5000) and Anti-rabbit IgG, HRP-linked Antibody (Cell Signaling Technology, Danvers, MA, USA, 1: 2000).

### 2.5. Cell Proliferation Assay

ESCC cells were cultured, and cell suspensions were prepared with medium containing 10% serum. Cells were counted at an initial density of 10000 cells per well and reinoculated in 96-well plates. The cells were incubated for 6 h until adherence. The medium was replaced with a freshly prepared medium containing NDP and added at the different concentration gradients shown. The cells were cultivated for 48 h and treated with the MTS assay. A BioTek ELx800 microplate reader (Bio-Tek Instruments, Winooski, VT, USA) was used to detect the absorbance of each well at 490 nm. The IC_50_ and inhibition ratio were calculated using GraphPad Prism 8.0 software (GraphPad Prism Software Inc., San Diego, CA, USA).

### 2.6. Colony Formation Assay

For examination of cell proliferation ability, colony formation assay was conducted [25, 26]. Transfected cells were first routinely digested and counted and further reseeded into 12-well plates at an initial density of 1000 (overexpression experiment group) or 4000 (knockdown experiment group) cells per well. Stop the cell culture when there are obvious cell clones visible to the naked eye. Calculate the clone formation rate after crystal violet staining (clone formation rate = the number of clones/number of inoculated cells × 100%).

### 2.7. Cell Viability Assay: MTS Assay

For examination of cell proliferation ability, MTS assay was conducted [25, 26]. Cells were reseeded into 96-well plates at an initial density of 6000 (overexpression experiment group) or 8000 (knockdown experiment group) cells per well. After 0 h, 24 h, 48 h, and 72 h, cell proliferation assays were performed using Cell Titer 96 Aqueous One Solution Cell Proliferation Assay Kit (Promega, Shanghai, China), also known as MTS assay.

### 2.8. Wound Healing Assay

For examination of cell migration ability, wound healing assay was conducted. For wound healing assay, inoculate the above-mentioned cells into a 12-well plate, 1 mL per well, and after adherence for 6 h, the cells were incubated in the medium supplemented with 2% FBS. Micrographs of the assigned areas were taken at 0 h and after a certain amount of time by the IX73 inverted microscope (Olympus, Tokyo, Japan). The areas of wound healing assay were analyzed by using ImageJ 1.52a (US National Institutes of Health, Bethesda, MD, USA).

### 2.9. Transwell Assay

For examination of cell migration ability, transwell assay was conducted. After cell counting of transfected cells, take 400 *μ*L cell suspension and add it to the transwell chamber. Inoculate onto the Falcon Chambers (BD Biosciences, Franklin Lakes, NJ, USA) with a density of 50000 (overexpression experiment group) or 60000 (knockdown experiment group) cells per well. After 48 h, the cells that migrated toward the lower chambers were stained with 0.5% crystal violet. Each assay was photographed for 5 views under the IX73 inverted microscope (Olympus, Tokyo, Japan), and the number of cells within each chamber was counted by ImageJ 1.52a (US National Institutes of Health, Bethesda, MD, USA).

### 2.10. Flow Cytometry Assay

Apoptosis was investigated by the Annexin V-FITC assay. Cells after siRNA or plasmids transfection were incubated with NDP for 48 h and washed by PBS twice. After incubation, cells were extracted and resuspended with Annexin V binding solution according to the C1062 Annexin V-FITC Apoptosis Detection Kit (Beyotime Biotechnology, Shanghai, China) instruction. Accuri C6 Plus (BD Biosciences, Franklin Lake, NJ, USA) was used to detect the apoptosis cells. The flow cytometry data for cell apoptosis was analyzed by FlowJo v. 7.6 software (Emerald Biotech Co., Ltd., Hangzhou, China).

### 2.11. Statistical Analysis

Statistics obtained from each assay were imported into GraphPad Prism 8 (GraphPad Prism Software Inc., San Diego, CA, USA) and SPSS 22.0 (SPSS Inc., Chicago, IL, USA) for graphing and analysis. All experimental results are presented as the mean ± SD. Student's *t*-tests were used to determine the statistical differences between independent samples. *P* value < 0.05 was defined as statistically significant.

## 3. Results

### 3.1. TRIP13 Is Abnormally Highly Expressed in ESCC

**Figure 1:**
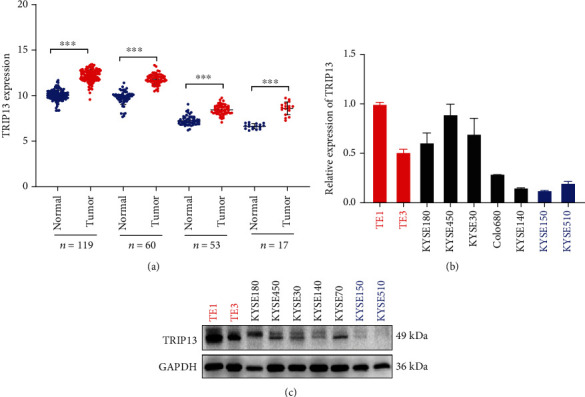
TRIP13 is high expressed in ESCC. (a) Data from Oncomine and GEO reveal that in the tumor tissues, TRIP13 is highly expressed. (b) TRIP13 mRNA expression level measured by qRT-PCR. (c) TRIP13 protein expression level measured by Western blot.

To assess the expression level of TRIP13 in ESCC, we analyzed several data sets. In the four data sets from Oncomine (https://www.oncomine.org/) and GEO (GSE53624, GSE20347), TRIP13 is highly expressed in cancerous tissue (Figure 1(a)).

### 3.2. High TRIP13 Expression Promotes Proliferation of ESCC

The effect of high TRIP13 expression in ESCC cells is definitely significant, which indicates a poor prognosis of ESCC patients [23]. We therefore evaluated the abilities of ESCC cells with abnormal TRIP13 protein expression. In our study, we firstly detected the mRNA and protein expression of TRIP13 in ESCC cells by qRT-PCR and Western blot. And TE1, TE3, and KYSE30 with relatively high expression of TRIP13 were chosen to knock down TRIP13 (Figures 1(b) and 1(c)), while KYSE150 and KYSE510 with relatively low expression of TRIP13 were chosen to conduct high TRIP13 expression (Figures 1(b) and 1(c)).

**Figure 2:**
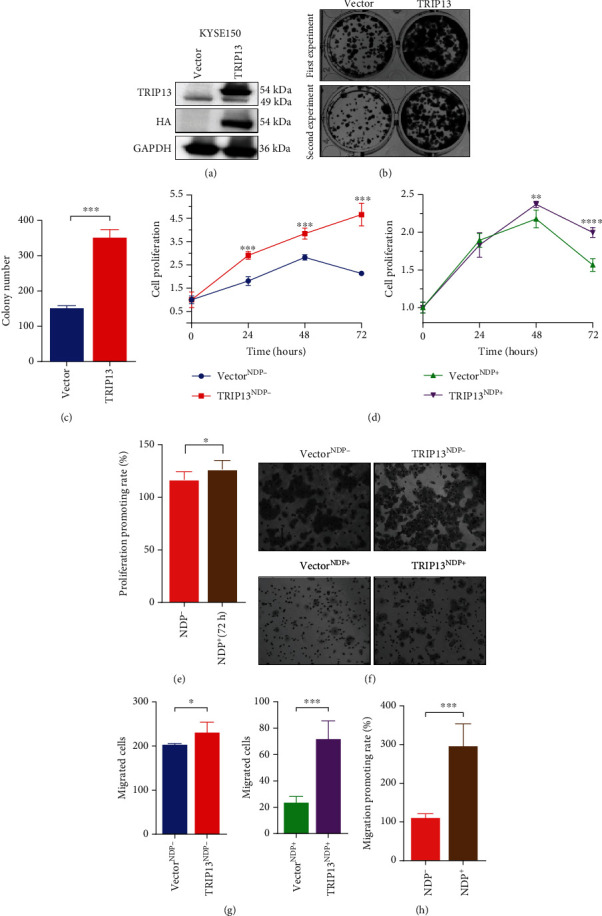
High TRIP13 expression promotes cell proliferation and migration as well as induces resistance to nedaplatin in KYSE150 cells. (a) The TRIP13 plasmids were transfected successfully into KYSE150 cells examined by Western blot. (b) Colony formation assay of KYSE150 with TRIP13 plasmids transfection. (c) The cell proliferation of KYSE150 was increased after TRIP13 plasmids transfection tested by colony formation assay. (d) With high expression of TRIP13 induced by TRIP13 plasmids, the cell proliferation of KYSE150 cells was increased tested by MTS assay. (e) With the effect of nedaplatin, the proliferation promoting rate in KYSE150 cells was enhanced. (f) Transwell assay of KYSE150 cells with TRIP13 plasmids transfection. (g) The cell migration was increased in KYSE150 cells after TRIP13 plasmids transfection. (h) With the effect of nedaplatin, the migration promoting rate in KYSE150 cells was enhanced.

**Figure 3:**
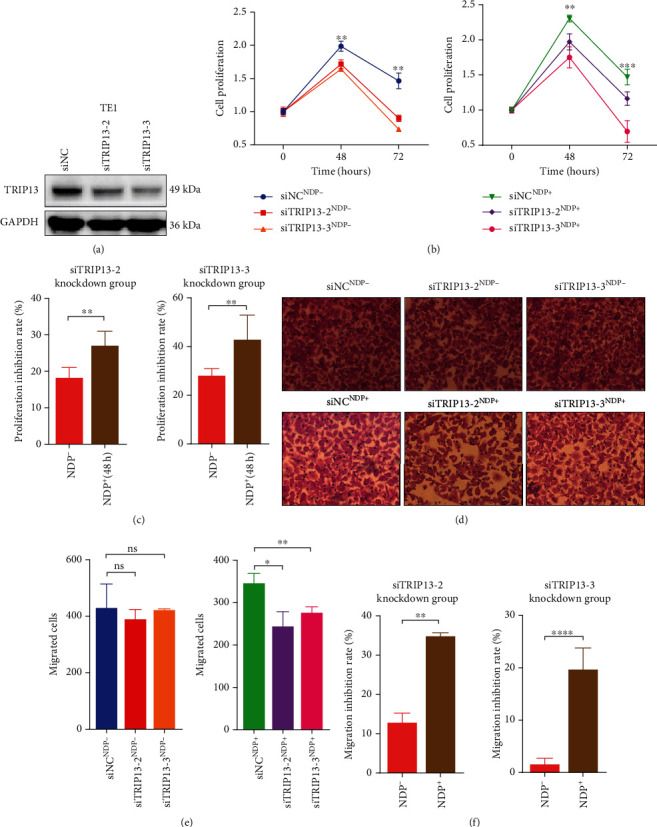
Low TRIP13 expression inhibits cell proliferation and migration as well as weakens resistance to nedaplatin in TE1 cells. (a) The siTRIP13-2 and siTRIP13-3 were transfected successfully into TE1 cells examined by Western blot. (b) The cell proliferation of TE1 cells was decreased after siTRIP13-2 and siTRIP13-3 transfection tested by MTS assay. (c) With the effect of nedaplatin, the proliferation inhibition rate of TE1 cells was enhanced. (d) Transwell assay of TE1 cells with siTRIP13-2 and siTRIP13-3 transfection. (e) The cell migration was decreased in TE1 cells after siTRIP13-2 and siTRIP13-3 transfection. (f) With the effect of nedaplatin, the migration inhibition rate of TE1 cells was enhanced.

**Figure 4:**
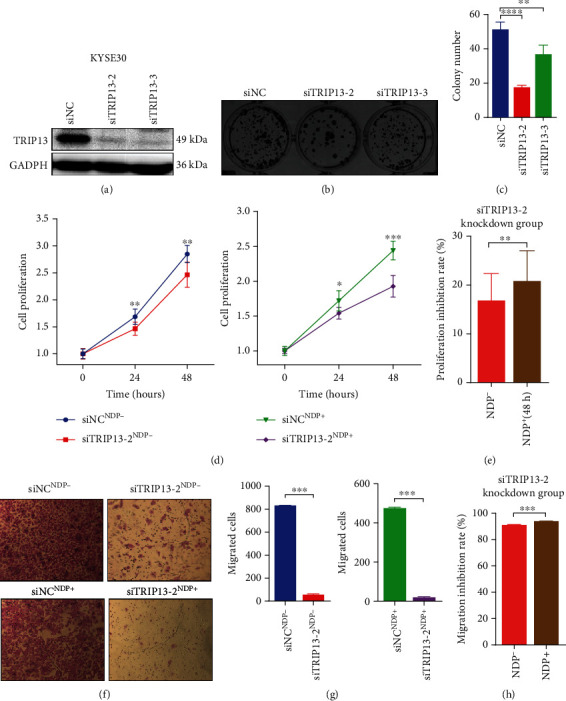
Low TRIP13 expression inhibits cell proliferation and migration as well as weakens resistance to nedaplatin in KYSE30 cells. (a) The siTRIP13-2 and siTRIP13-3 were transfected successfully into KYSE30 cells examined by Western blot. (b) Colony formation assay of KYSE30 with siTRIP13-2 and siTRIP13-3 transfection. (c) The cell proliferation of KYSE30 was decreased after siTRIP13-2 and siTRIP13-3 transfection tested by colony formation assay. (d) The cell proliferation of KYSE30 cells was decreased after siTRIP13-2 transfection tested by MTS assay. (e) With the effect of nedaplatin, the proliferation inhibition rate of KYSE30 cells was enhanced. (f) Transwell assay of KYSE30 cells with siTRIP13-2 transfection. (g) The cell migration was decreased in KYSE30 cells after siTRIP13-2 transfection. (h) With the effect of nedaplatin, the migration inhibition rate of KYSE30 cells was enhanced.

To evaluate the effect of TRIP13 in proliferation ability of ESCC, we transfected the TRIP13 siRNA and TRIP13 expression plasmid into ESCC cells. And the transfection efficiency was assessed by Western blot (Figures 2(a), 3(a), and 4(a), Supplementary Fig. 1a, Supplementary Fig. 2a). In addition, the MTS assay and colony formation assay were used to detect the proliferation ability of ESCC cells. From the results, KYSE150 with markedly upregulated TRIP13 protein had a great improvement in the proliferation ability (Figures 2(b)–2(d)), whereas inhibition of proliferation ability was observed in TE1 and KYSE30 with significant decreased TRIP13 protein (Figures 3(b) and 4(b)–4(d)).

### 3.3. High TRIP13 Expression Promotes Migration of ESCC

Apart from the proliferation ability of ESCC cells with abnormal TRIP13 protein expression, figuring out the migration ability of it is equally essential. Transwell assay and wound healing assay were used to investigate the role of TRIP13 in migration ability of ESCC. The transfection efficiency of TRIP13 expression was confirmed in Western blot (Figures 2(a), 3(a), and 4(a), Supplementary Fig. 1a, Supplementary Fig. 2a). The results displayed that KYSE150 and KYSE510 with high TRIP13 expression promoted the migrating ability compared with the control group (Figures 2(f) and 2(g), Supplementary Fig. 1b, Supplementary Fig. 1c, Supplementary Fig 1e, Supplementary Fig 1f) and TE1, KYSE30, and TE3 with low TRIP13 expression inhibited the migrating ability compared with the control group (Figures 3(d), 3(e), 4(f), and 4(g), Supplementary Fig. 2b, Supplementary Fig. 2c).

### 3.4. High TRIP13 Expression Induces NDP Resistance in ESCC

Although NDP has a good antitumor effect on ESCC [27–29], it is unknown whether ESCC is resistant to it. In addition, previous studies have showed that TRIP13 induces platinum resistance in carcinoma, including head and neck squamous cell carcinoma and bladder cancer [21, 22]. Therefore, we investigated the effect of TRIP13 on NDP resistance in ESCC assessed by cell function experiment. With NDP intervention, MTS assay was used to examine the proliferation of ESCC, including KYSE150, TE1, and KYSE30, after TRIP13 overexpression or knockdown. Also, transwell assay and wound healing assay was used to examine the migration of ESCC, including KYSE150, TE1, KYSE30, KYSE510, and TE3 after TRIP13 knockdown or overexpression. We analyzed the cell proliferation promoting/inhibition rate and cell migration promoting/inhibition rate. The results showed that NDP had a weaker inhibitory effect on the cells after the TRIP13 overexpression compared with the control group (Figures 2(e) and 2(h), Supplementary Fig. 1d, Supplementary Fig. 1g) while it had a greater inhibitory effect on the cells after the TRIP13 knockdown (Figures 3(c), 3(f), 4(e), and 4(h), Supplementary Fig. 2d). Taken together, the cells after TRIP13 overexpression were less sensitive to NDP while the cells after TRIP13 knockdown were more sensitive to NDP, revealing that high TRIP13 expression led the cell resistance to NDP.

### 3.5. High TRIP13 Expression Induces NDP Resistance via Enhancing Repair of DNA Damage and Inhibiting Cell Apoptosis in ESCC

**Figure 5:**
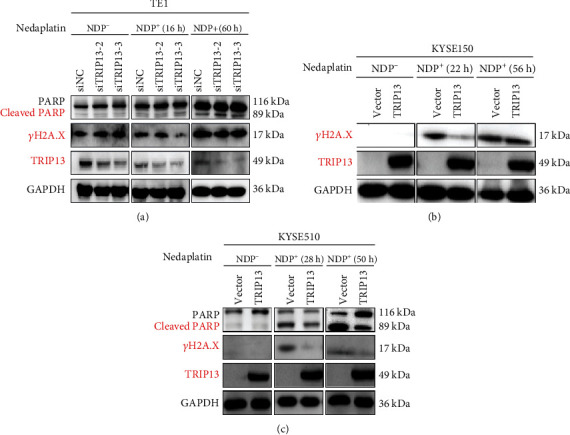
High TRIP13 expression induces NDP resistance via decreasing PARP and *γ*H2A.X expression in ESCC. (a) The cleaved PARP expression level of the TRIP13 knockdown group increased with the effect of nedaplatin in TE1 cells examined by Western blot. Also, the *γ*H2A.X expression level increased after the effect of nedaplatin examined by Western blot. (b) The *γ*H2A.X expression level of the TRIP13 overexpression group was lower in KYSE150 cells tested by Western blot. (c) The cleaved PARP expression level of the TRIP13 overexpression group decreased with the effect of nedaplatin in KYSE510 cells examined by Western blot. Also, the *γ*H2A.X expression level decreased after the effect of nedaplatin examined by Western blot.

**Figure 6:**
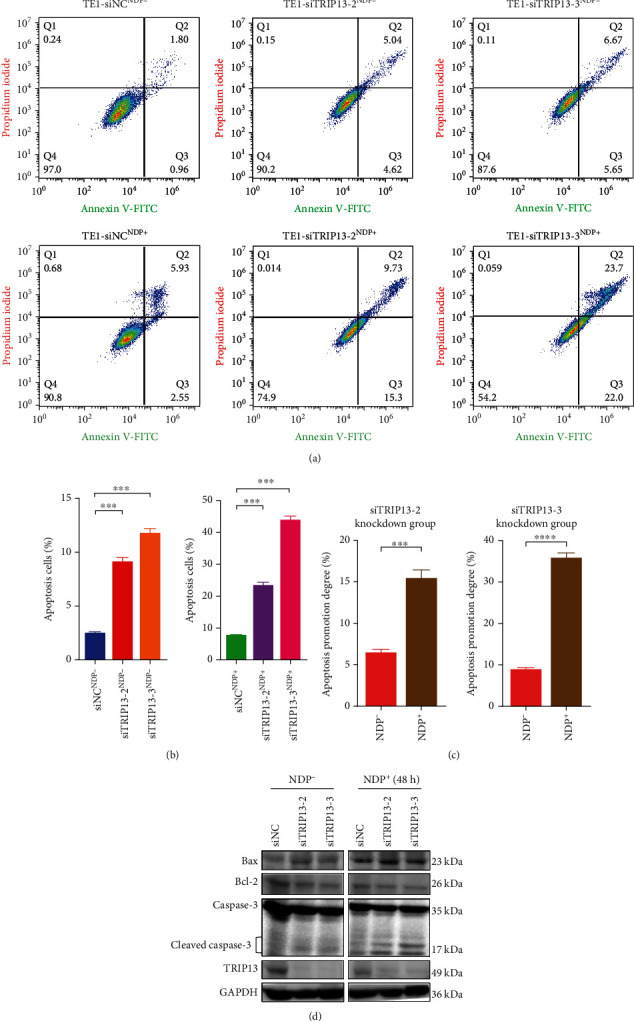
Low TRIP13 expression inhibits NDP resistance via increasing apoptotic cells, increasing the expression of proapoptotic protein and decreasing the expression of antiapoptotic protein in ESCC. (a, b) The proportion of apoptotic cells increased in TE1 cells treated with TRIP13 knockdown and NDP for 48 h tested by flow cytometry. (c) The apoptosis promotion degree was enhanced in TRIP13 knockdown TE1 cells with DNP treatment. (d) The expression of cleaved caspase-3 and Bax increased, and the expression of Bcl-2 decreased in TE1 cells with TRIP13 knockdown and NDP intervention tested by Western blot.

**Figure 7:**
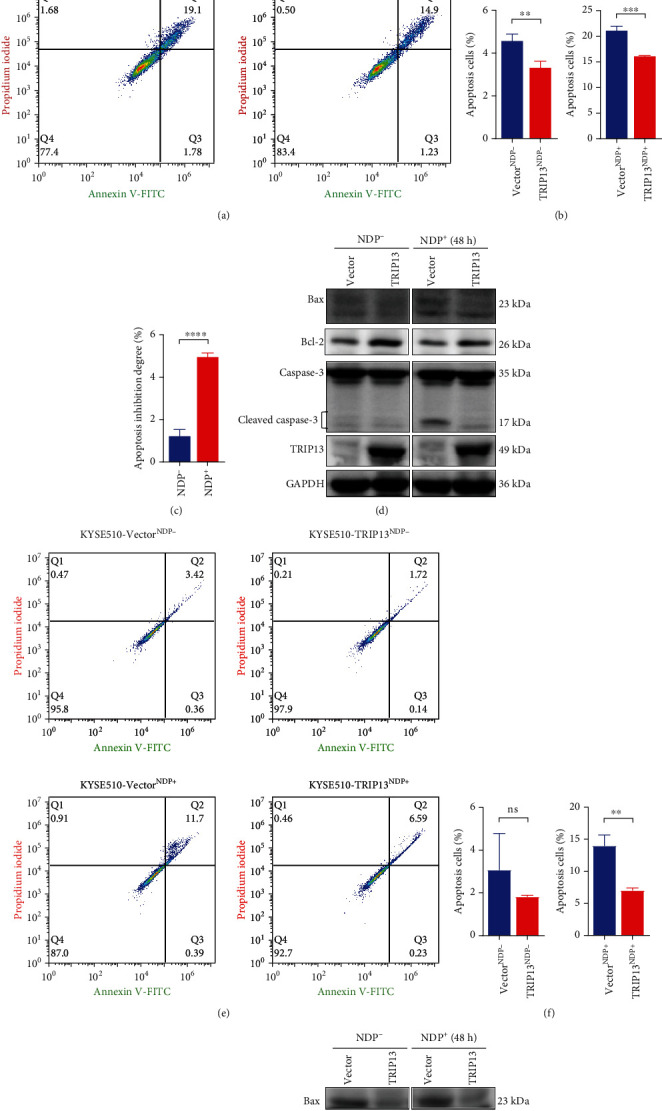
High TRIP13 expression induces NDP resistance via decreasing apoptotic cells, decreasing the expression of proapoptotic protein and increasing the expression of anti-apoptotic protein in ESCC. (a, b, e, f) The proportion of apoptotic cells decreased in KYSE150 and KYSE510 cells treated with TRIP13 overexpression and NDP for 48 h tested by flow cytometry. (c, g) The apoptosis inhibition degree was reduced in TRIP13 overexpression KSYE150 and KYSE510 cells with DNP treatment. (d, h) The expression of cleaved caspase-3 and Bax decreased, and the expression of Bcl-2 increased in KYSE150 and KYSE510 cells with TRIP13 overexpression and NDP intervention tested by Western blot.

PARP is a marker of DNA damage repair process [30]. As is shown in Figures 5(a) and 5(c), with NDP intervention, PARP and cleaved PARP expression increased in TE1 cell and KYSE510 cell, indicating the existence of DNA damage and repair in ESCC. In addition, compared with the control group, increased cleaved PARP expression in the TRIP13 knockdown group (Figure 5(a)), indicated decreased TRIP13 expression contributed to increased DNA damage and sensitivity to NDP in TE1 cells. On the contrary, in Figure 5(c), the cleaved PARP expression in the high TRIP13 expression group was lower than that of the control group with the effect of NDP, revealing that increased TRIP13 expression contributed to decreased DNA damage and less sensitivity to NDP in KYSE510 cells. H2A.X is a marker of cell apoptosis [31]. In Figures 5(a) and 5(c), the cell apoptosis expression in TE1 and KYSE510 cells increased due to the inhibition of NDP. Compared with the control group, the *γ*H2A.X expression level of TRIP13 overexpression group was lower in KYSE150 cell and KYSE510 cell (Figures 5(b) and 5(c)), which demonstrated that when TRIP13 overexpressed, the cell apoptosis would decrease, and therefore, the NDP drug effect to ESCC would decrease. In order to further figure out the mechanism of TRIP13 overexpression inducing NDP resistance, we performed flow cytometry assay and detected the expression of apoptosis-related proteins. Compared with the control group, the proportion of apoptotic cells increased in TE1 cells after TRIP13 knockdown (Figures 6(a) and 6(b)). After NDP treatment, the apoptosis of TE1 cells with TRIP13 knockdown was more obvious (Figure 6(c)). Some members of caspase family also participate in the process of apoptosis. When the apoptosis process increased, the expression of cleaved caspase-3 increased in TE1 cells with TRIP13 knockdown (Figure 6(d)). After NDP intervention, the apoptosis is more serious (Figure 6(d)). We also detected the expression of Bcl-2 and Bax. The results showed that the expression of Bcl-2 decreased and the expression of Bax increased after TRIP13 depletion and NDP treatment, indicating that the apoptotic activity of TE1 cells increased (Figure 6(d)). On the contrary, compared with the control group, the proportion of apoptotic cells in KYSE150 cells and KYSE510 cells after TRIP13 overexpression was significantly reduced (Figures 7(a), 7(b), 7(e), and 7(f)). After NDP treatment, the apoptosis of KYSE150 cells and KYSE510 cells with TRIP13 overexpression weakened (Figures 7(c) and 7(g)). Also, the expression of cleaved caspase-3 and Bax decreased, and the expression of Bcl-2 increased (Figures 7(d) and 7(h)). To sum up, high TRIP13 expression induces NDP resistance via enhancing repair of DNA damage and inhibiting cell apoptosis in ESCC.

## 4. Discussion

ESCC is one of the common malignant tumors with high level of incidence and mortality, whose incidence ranked seventh and the mortality ranked sixth in the world in 2018 [1]. The treatments to ESCC include surgery, chemotherapy, and radiotherapy [1, 9, 32, 33]. At present, chemotherapy and chemoradiotherapy still plays important roles in adjuvant treatments [34].

In chemotherapy or chemoradiotherapy, platinum-based drugs are commonly used, among which NDP has lower toxicity except bone marrow toxicity compared with cisplatin [35]. The mechanism of cisplatin is to influence the structure of DNA double helix by forming platinum-DNA adducts, which leads to replication, transcription inhibition and DNA double strand breaks (DSB), thus activating signal transduction pathway and initiating DNA repair [8, 36, 37]. Failure of DNA repair or excessive damage will lead to apoptosis and achieve chemotherapy effect [8, 36, 37]. The increase of DNA repair process is considered to be the most prominent feature of platinum-resistant cells, but the defect of DNA mismatch repair will lead to the enhancement of cisplatin resistance [36]. The recognition of platinum-DNA adducts involves several protein families, including nonhistone chromosome high-mobility group proteins 1 and 2 (HMG1 and HMG2), nucleotide excision repair (NER) protein, and mismatch repair (MMR) protein [8]. One of the mechanisms by which cancer cells become resistant to cisplatin is derived from changes in any of these molecular circuits [38]. PARP (poly(ADP-ribosyl)ated proteins) is considered as a marker of DNA damage repair, which is mainly involved in base excision repair [8]. PARP starts repairing cells when DNA was damaged. If repair fails, PARP will be sheared to become cleaved PARP [30]. PARP is associated with the regulation of MMR and NER, which can promote DSB repair, and it is highly expressed in cisplatin-resistant cells [8, 39]. PARP is also one of the downstream substrates of the caspase family, which plays a vital role in cell apoptosis [40]. As a member of the caspase family, caspase-3 can activate procaspase-3, activate PARP, and therefore lead to cell apoptosis [40]. Bcl-2 family members are a group of important regulatory factors in apoptosis activities [41]. They are divided into antiapoptotic proteins and proapoptotic proteins [41]. Bax is a proapoptotic protein, initially located in the cytoplasm, and can be transferred to the mitochondrial outer membrane for subsequent conformational changes after the initiation of apoptosis [41]. Through the regulation of cytochrome c, AIF, and other factors, Bcl-2 family proteins can indirectly coordinate the activity of caspase proteins in apoptotic pathways [42, 43]. Meanwhile, DSB triggers the expression of *γ*H2A.X. H2A.X is a marker of apoptosis. When apoptosis occurs, H2A.X is phosphorylated into *γ*H2A.X. When the cell repair fails, *γ*H2A.X is unable to dephosphorylate and reverts to H2A.X, resulting in the upregulation of *γ*H2A.X expression [31, 44].

Nedaplatin, as the second generation platinum anticancer drug with a favorable clinical effect [45, 46], is a derivative of cisplatin, and its anticancer molecular mechanism is similar to that of cisplatin [47]. However, research proved that multiple cancers present resistance to NDP, including nasopharyngeal carcinoma, cervical cancer, and non-small-cell lung cancers [13–15].

TRIP13 gene, a member of the AAA+ ATPase super-family, is located in 5p15.33, encoding TRIP13 protein, which is mainly involved in cell mitosis and repair of DNA damage [48–50]. Research revealed that the abnormal expression of TRIP13 indicated a poor prognosis of cancer patients [20, 23]. A study displayed that knocking down TRIP13 can inhibit the cell proliferation of human chronic lymphocytic leukemia [19]. In addition, a study showed that loss of TRIP13 in tubular epithelial cell is more likely to apoptosis [51]. In our study, we proved that high expression of TRIP13 could increase the proliferation and migration of ESCC cells while knocking down TRIP13 inhibited the cell proliferation and migration. With the effect of NDP, high TRIP13 expression could also enhance the cell proliferation as well as cell migration of ESCC cells while reduced TRIP13 expression inhibited the cell proliferation as well as cell migration. Therefore, these findings indicated that high expression of TRIP13 enhanced the proliferation and migration ability of ESCC cells.

In the previous research, TRIP13 could promote drug resistance to head and neck squamous cell carcinoma by enhancing the repair effect of DNA damage [21]. Moreover, high expression of TRIP13 could lead to cisplatin and doxorubicin resistance in bladder cancer [22]. These findings reminded us that TRIP13 may be a factor leading to drug resistance in cancer cells. In this study, we found that with the NDP intervention, the proliferation/migration promoting rate was increased, indicating that high TRIP13 expression of ESCC cells had stronger resistance to NDP. On the contrary, the proliferation/migration inhibition rate was increased after the effect of NDP, revealing that the TRIP13-deficient ESCC cells were more sensitive to NDP. After knocking down TRIP13, ESCC cells were more likely to conduct apoptosis. These results indicated that ESCC cells with high TRIP13 expression are easier to resist NDP and ESCC cells are more likely to be alive.

Several studies have suggested that the molecular mechanism of drug resistance to cancer cells is different [13–15, 21]. Homologous recombination (HR) and nonhomologous end joining (NHEJ) are common repair processes of DSB [52]. Researchers found that TRIP13 could promote the DSB repair of head and neck squamous cell carcinoma cells through NHEJ, resulting in drug resistance [21]. Another study showed that with high expression of TRIP13, the expression of *γ*H2A.X decreased and the expression of RAD50 increased in bladder cancer cells after cisplatin treatment, which revealed that TRIP13 promoted DSB repair and reduced apoptosis of cancer cells treated with drugs [22]. Since platinum drugs cause cytotoxicity by promoting DNA damage in cells [53], in our study, we investigated the expression of apoptosis-related proteins. We found that with NDP treatment, the expression of Bcl-2 increased and PARP, *γ*H2A.X, cleaved caspase-3, and Bax expression decreased when the expression of TRIP13 was high. In contrast, the expression of Bcl-2 decreased and PARP, *γ*H2A.X, cleaved caspase-3, and Bax expression increased when knocking down TRIP13. These results suggested that high TRIP13 expression-induced NDP resistance made ESCC cells easier to escape the toxicity of NDP. Therefore, ESCC cells with high TRIP13 expression exists NDP resistance may be through increasing repair of DNA damage and decreasing apoptosis. Based on the previous studies and our results, DSB repair of ESCC with high TRIP13 expression may be caused by the promotion of NHEJ. TRIP13 generally promotes the progression of tumor cells by affecting cell cycle [48, 54]. DNA double strands are formed during cell cycle, and nedaplatin inhibits the development of tumor cells by disrupting the structure of DNA double strands. The results showed that high expression of TRIP13 can reduce the ability of nedaplatin to inhibit tumor cell development. TRIP13 and nedaplatin present a certain role in the cell cycle progress. Therefore, the resistance of ESCC to nedaplatin may also be related to cell cycle changes.

## 5. Conclusion

In conclusion, high expression of TRIP13 can promote the proliferation and migration ability of ESCC cells, which contributes to the resistance effect to the NDP. And the molecular mechanisms of the NDP resistance may be through increasing repair of DNA damage and decreasing apoptosis. However, more detailed mechanisms are needed to be investigated, which may provide more evidence for the therapeutic usage of NDP in the clinical situation.

## Figures and Tables

**Table 1:** TRIP13 siRNA sequence.

Catalog number	Gene name	Sequence (5′-3′)	
Negative control (siNC)	*TRIP13* (human)	UUC UCC GAA CGU GUC ACG UTT	ACG UGA CAC GUU CGG AGA ATT
TRIP13-Homo-404 (siTRIP13-2)	*TRIP13* (human)	CCC AUC GAU UUG AGU GCA UTT	AUG CAC UCA AAU CGA UGG GTT
TRIP13-Homo-533 (siTRIP13-3)	*TRIP13* (human)	GCU GAA UUC CAU GGG CUU UTT	AAA GCC CAU GGA AUU CAG CTT

## Data Availability

The data used to support the findings of this study are available from the corresponding author upon request.

## Supplementary Materials

Supplementary 1 Supplementary Fig. 1: high TRIP13 expression promotes cell migration and induces resistance to nedaplatin in KYSE510 cells.

Supplementary 2 Supplementary Fig. 2: low TRIP13 expression inhibits cell migration and weakens resistance to nedaplatin in TE3 cells.

Supplementary 3 Supplementary Table 1: primers used in this study.
