# Supplementary material for: TRIP13 Induces Nedaplatin Resistance in Esophageal Squamous Cell Carcinoma by Enhancing Repair of DNA Damage and Inhibiting Apoptosis
Source: Biomed Res Int. 2022 May 10;2022:7295458. doi: 10.1155/2022/7295458 (PMC9115607; doi:10.1155/2022/7295458)
Supplement: Supplementary 2 — Supplementary Fig. 2: low TRIP13 expression inhibits cell migration and weakens resistance to nedaplatin in TE3 cells. [file 7295458.f2.docx]

**Article title:** TRIP13 induces nedaplatin resistance in esophageal squamous cell carcinoma by enhancing repair of DNA damage and inhibiting apoptosis

**Journal name:** Biomed Research International

**Author names:** Lin-Ting Zhang, Li-Xin Ke, Xin-Yi Wu, Hui-Ting Tian, Hua-Zhen Deng, Li-Yan Xu, En-Min Li, Lin Long

**Affiliation of the corresponding author:** The Key Laboratory of Molecular Biology for High Cancer Incidence Coastal Chaoshan Area, Shantou University Medical College, Shantou 515041, Guangdong Province, China

**E-mail address of the corresponding author:** llong@stu.edu.cn

**Supplementary Figure 2**


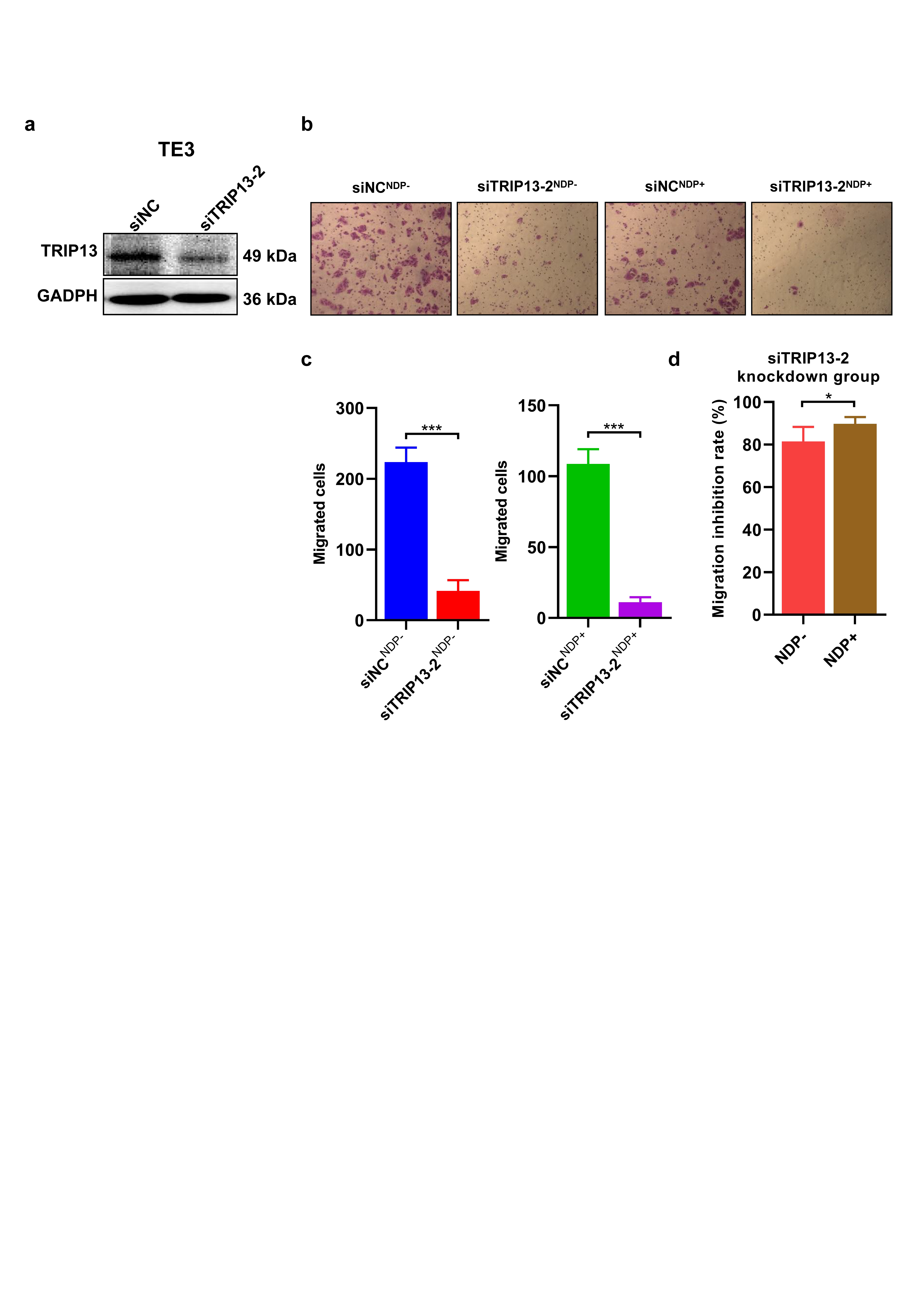


**Supplementary Fig. 2 Low TRIP13 expression inhibits migration rates as well as weakens resistance to nedaplatin in TE3 cells**

**(a)** The siTRIP13-2 was transfected successfully into TE3 cells examined by Western blot. **(b)** Transwell assay of TE3 cells with siTRIP13-2 transfection. **(c)** The migration rate was decreased in TE3 cells after siTRIP13-2 transfection. **(d)** With the effect of nedaplatin, the migration inhibition rate of TE3 cells was enhanced.
