# Supplementary material for: TRIP13 Induces Nedaplatin Resistance in Esophageal Squamous Cell Carcinoma by Enhancing Repair of DNA Damage and Inhibiting Apoptosis
Source: Biomed Res Int. 2022 May 10;2022:7295458. doi: 10.1155/2022/7295458 (PMC9115607; doi:10.1155/2022/7295458)
Supplement: Supplementary 3 — Supplementary Table 1: primers used in this study. [file 7295458.f3.docx]

**Article title:** TRIP13 induces nedaplatin resistance in esophageal squamous cell carcinoma by enhancing repair of DNA damage and inhibiting apoptosis

**Journal name:** Biomed Research International

**Author names:** Lin-Ting Zhang, Li-Xin Ke, Xin-Yi Wu, Hui-Ting Tian, Hua-Zhen Deng, Li-Yan Xu, En-Min Li, Lin Long

**Affiliation of the corresponding author:** The Key Laboratory of Molecular Biology for High Cancer Incidence Coastal Chaoshan Area, Shantou University Medical College, Shantou 515041, Guangdong Province, China

**E-mail address of the corresponding author:** llong@stu.edu.cn

**Supplementary Table 1 Primers used in this study**

| **Primer name** | **Sequence (5′-3′)** |
| --- | --- |
| **Primers for cloning** | |
| TRIP13 (HA) - F | GACTCAGATCTCGAGGCCACCATGGACGAGGCCGTGGGCGACCTG |
| TRIP13 (HA) - R | GCCGCTGCAGAATTCGATGTAAGCTGCAAGCTTCTT |
| TRIP13 (GFP) - F | GACTCAGATCTCGAGCCATGGACGAGGCCGTGGGCGACCTG |
| TRIP13 (GFP) - R | TCGACTGCAGAATTCTCAGATGTAAGCTGCAAGCTTCTT |
| **Primers for qRT-PCR** | |
| TRIP13 - F | CTGTCTCTGGCAGTGGACAAG |
| TRIP13 - R | TTGGTTTGCAGAAGGGATTC |
| β-actin - F | CAACTGGGACGACATGGAGAAA |
| β-actin - R | GATAGCAACGTACATGGCTGGG |
